# Multi-Scale Bionic Materials: Interfacial Design, Effective Fabrication and Functional Application

**DOI:** 10.3390/ma19122569

**Published:** 2026-06-14

**Authors:** Haoqi Yang

**Affiliations:** College of Electrical, Energy and Power Engineering, Institute of Technology for Carbon Neutralization, Yangzhou University, Yangzhou 225127, China; yanghq@yzu.edu.cn

## 1. Introduction and Scope

Bionic materials represent an important frontier in modern materials science, where structural motifs, interfacial mechanisms, and functional strategies derived from natural systems are translated into engineered materials with enhanced performance [1,2,3]. Over their long-term evolution, biological materials have developed sophisticated architectures across molecular, nano-, micro-, meso-, and macroscopic scales. These architectures enable natural systems to integrate seemingly conflicting properties, such as stiffness and toughness, adhesion and reversibility, permeability and selectivity, a lightweight structure and mechanical robustness, or biological activity and environmental adaptability [4,5]. Typical examples include the layered toughening interfaces of nacre, the hierarchical mineral–organic architecture of bone, the wet adhesion of barnacle cement and mussel proteins, the directional channels of wood, the anti-freezing behavior of biological proteins, and the functional surfaces of lotus leaves, spider silk, and fish scales [6,7,8,9]. These natural prototypes provide abundant inspiration for the rational design of advanced materials with multifunctional and adaptive characteristics.

The development of multi-scale bionic materials is no longer limited to the direct imitation of biological morphology. Instead, it increasingly emphasizes the extraction of underlying design principles from nature and their integration into material composition, interface regulation, structural construction, and functional application. Among these principles, interfacial design plays a central role. Interfaces determine how different components interact, how stress and energy are transferred or dissipated, how ions or molecules migrate, and how cells, microorganisms, or tissues respond to material surfaces [10,11,12]. By learning from natural interfacial systems, researchers can construct materials with improved adhesion, toughness, self-healing abilities, biocompatibility, ion selectivity, antibacterial activity, and environmental stability. Effective fabrication is another key issue in this field. The complex architectures of biological materials usually involve precise structural control over multiple length scales, which cannot be fully reproduced by conventional processing methods. Advanced fabrication technologies, including additive manufacturing, electrospinning, laser-assisted deposition, controlled polymerization, self-assembly, foam templating, and post-synthetic modification, provide powerful tools for constructing bionic materials with controllable geometry, porosity, surface chemistry, and hierarchical organization [13,14]. These methods enable researchers to bridge the gap between biological inspiration and practical materials engineering. Therefore, the functional applications of multi-scale bionic materials are broad and rapidly expanding. In biomedical engineering, bionic materials are being developed for bone repair, implant functionalization, tissue engineering, drug delivery, hemostasis, antibacterial protection, and medical adhesives. In sustainable materials and packaging, bioactive polymers, natural-product-functionalized films, nanofibrous membranes, and microbial cellulose scaffolds offer new opportunities for antibacterial, antioxidant, biodegradable, and environmentally friendly systems. In energy storage and conversion, biomimetic electrolyte engineering, artificial ion channels, antifreeze-protein-inspired additives, and bioinspired interfacial layers provide new strategies to address ion transport, dendrite growth, parasitic reactions, and low-temperature operation in aqueous batteries. In extreme-environment devices, bioinspired ceramics and structural coatings can improve high-temperature insulation, adhesion, and service stability. These examples demonstrate that multi-scale bionic materials are becoming a cross-disciplinary platform connecting materials science, chemistry, biology, medicine, energy, manufacturing, and sustainability.

The Special Issue “Multi-Scale Bionic Materials: Interfacial Design, Effective Fabrication and Functional Application” was organized to provide a focused platform for presenting recent advances in this rapidly developing field. The collected articles cover a wide range of topics, including bionic biomedical interfaces, bone-regeneration scaffolds, wet adhesives, bioactive coatings, antibacterial materials, functional polymer nanocarriers, microbial scaffolds, stimuli-responsive soft matter, sustainable packaging materials, biomimetic electrolyte engineering, and high-temperature functional ceramics. Together, these contributions highlight how lessons learned from natural systems can be transformed into advanced materials through rational interfacial design, effective fabrication, and functional integration. This Special Issue therefore aims to promote interdisciplinary communication and provide useful insights for the future development of multi-scale bionic materials.

## 2. Overview of Published Articles

This Special Issue includes eleven research papers and three review papers by authors from China, Romania, Poland, Taiwan, the Republic of Korea, the United States, Chile, and Indonesia. These contributions cover a broad range of topics related to multi-scale bionic materials, including bioinspired structural ceramics, biomedical coatings, bone-regeneration scaffolds, wet-adhesion biomaterials, conductive hydrogels, active packaging films, microbial cellulose scaffolds, polymer nanocarriers, biomimetic electrolyte engineering, and sustainable functional materials.

### 2.1. Bioinspired Biomedical Interfaces and Bone-Regeneration Materials

In the field of biomedical interface design and implant functionalization, Contribution 1 developed bioactive glass and melittin composite thin films for titanium implant surfaces using matrix-assisted pulsed laser evaporation. The obtained coatings improved surface wettability, corrosion resistance, and in vitro mineralization ability, indicating their potential for enhancing bone–implant integration. Contribution 2 focused on PEEK interspinous spacers for spinal fusion applications. By combining biomechanical compression testing, Raman spectroscopy, and microscopic analysis, this study clarified the influence of material type and manufacturing route on load distribution, deformation behavior, and implant reliability. Contribution 3 introduced star-polymer-stabilized silver nanoparticles into PMMA-based bone cement. The modified bone cement maintained acceptable compressive strength and exhibited significant antibacterial activity against *E. coli*, providing a feasible route for infection-resistant orthopedic implants.

In the area of bone-regeneration scaffolds and osteoinductive materials, Contribution 4 reviewed the state of the art and future prospects of biocompatible thermoplastics in the additive manufacturing of bone defect fillers. This review summarized the characteristics of PCL, PLA, PEEK, PMMA, and their ceramic composites, emphasizing the role of 3D printing in personalized scaffold fabrication. Contribution 5 synthesized nanosized lithium-modified calcium–organic frameworks and demonstrated their ability to promote apatite formation, support pre-osteoblast adhesion and viability, and enhance osteogenic differentiation. Contribution 6 designed and fabricated 3D-printed bionic hydroxyapatite ceramic scaffolds with BCC, FCC, and TPMS structures at different porosities. The results showed that the TPMS structure, especially at 80 vol.% porosity, achieved a favorable balance between compressive strength and biocompatibility, providing useful guidance for bionic bone scaffold design.

### 2.2. Sustainable, Antibacterial, and Biohybrid Functional Materials

Contribution 7 prepared electrospun polyvinylidene fluoride nanofibers enriched with tannic acid for active food packaging. The incorporation of tannic acid endowed the nanofiber membranes with antifungal, antibiofilm, antioxidant, and food-preservation functions, effectively improving the shelf life of cherry tomatoes. Contribution 8 developed biodegradable carboxymethyl cellulose-based films containing liquid products of pine wood pyrolysis. The resulting films showed significantly enhanced antioxidant activity, strong antibacterial effects against several pathogenic bacteria, and good biodegradability in soil, indicating their potential for sustainable packaging and biocidal material applications.

Contribution 9 constructed fungus-immobilized foamed bacterial cellulose scaffolds using *Pleurotus ostreatus* and *Aspergillus oryzae*. The foamed bacterial cellulose served as a three-dimensional porous matrix for fungal immobilization, maintaining high water content and swelling ability. SEM observations confirmed the integration of fungal-derived fibers with the cellulose network, while thermogravimetric analysis suggested improved thermal stability. This study demonstrates that microbial immobilization and bacterial cellulose biofabrication can be combined to construct biodegradable and functional biohybrid scaffolds for biotechnology, bioremediation, functional packaging, and sustainable materials.

### 2.3. Dynamic Soft Materials and Biomimetic Wet-Adhesion Interfaces

Contribution 10 reported a CO_2_-responsive worm-like micelle system based on a double-tailed surfactant. Under CO_2_ stimulation, the surfactant molecules underwent protonation and self-assembled into ultra-long-chain cationic worm-like micelles, resulting in a remarkable viscosity increase. The system showed reversible switching between high- and low-viscosity states under CO_2_/air stimulation, demonstrating its potential for smart fluids and stimuli-responsive soft materials. Contribution 11 investigated the effect of metal ions on alginate/polyacrylamide conductive hydrogels. Through dynamic metal–ligand coordination, especially Al^3+^ crosslinking, the hydrogel achieved improved tensile strength, conductivity, and self-healing ability, highlighting the importance of reversible coordination interfaces in soft bionic materials.

Contribution 12 reviewed recent advances in barnacle-inspired biomaterials in biomedical research. This review summarized the composition, self-assembly behavior, adhesion mechanism, and curing process of barnacle cement proteins, as well as their applications in medical adhesives, tissue engineering, drug delivery, and hemostasis. The authors emphasized that barnacle cement proteins can form stable nanofibers and exhibit strong underwater adhesion, water resistance, biocompatibility, and antibacterial potential. This contribution provides important mechanistic inspiration for the design of next-generation wet adhesives and biomedical interfacial materials.

### 2.4. Energy Storage and Extreme-Environment Functional Applications

Contribution 13 reviewed biomimetic engineering strategies in electrolytes for aqueous batteries. The authors proposed a unified framework involving SEI–mimetic, antifreeze-protein–mimetic, and ion-channel–mimetic strategies, corresponding to water activity regulation, interfacial mechanics, and sub-nanometer ion transport. This review covered aqueous monovalent-ion batteries, multivalent-ion batteries, and redox-flow batteries, showing how biological membranes, antifreeze proteins, ion channels, wood-like structures, and natural polymer networks can inspire electrolyte and interface design for safe, low-cost, and high-performance aqueous energy storage systems.

Contribution 14 prepared bioinspired structural ceramics for application in K-type coaxial thermocouples. Inspired by the needle-like crystals in jujube leaves, the authors designed a ceramic insulating layer with high-temperature electrical insulation and strong adhesion. The ceramic layer showed stable insulation and adhesion under high-temperature conditions, and the integrated thermocouple exhibited reliable temperature-measurement performance under flame and laser thermal-shock environments. This work expands the application of multi-scale bionic materials from biomedical and soft-material systems to extreme-environment sensing and aerospace-related devices.

## 3. Conclusions

This Special Issue provides a comprehensive overview of recent advances in multi-scale bionic materials, with contributions ranging from bioinspired biomedical interfaces, bone-regeneration scaffolds, wet-adhesion biomaterials, conductive and self-healing hydrogels, sustainable antibacterial packaging materials, microbial biohybrid scaffolds, and polymer nanocarriers to biomimetic electrolytes and high-temperature functional ceramics. More specifically, the fourteen peer-reviewed contributions collected in this Special Issue demonstrate that bionic materials are moving beyond simple morphological imitation toward a deeper integration of biological principles, interfacial mechanisms, advanced fabrication, and functional applications. These studies reveal how natural design strategies, such as hierarchical bone architecture, barnacle wet adhesion, dynamic coordination networks, microbial cellulose scaffolds, ion-channel transport, antifreeze regulation, and plant- or biomass-derived bioactivity, can inspire the rational development of advanced materials with improved mechanical performance, biocompatibility, antibacterial activity, ion transport regulation, environmental adaptability, and service reliability. At the same time, this Special Issue also highlights several key challenges that should be addressed in future research, including the precise correlation between biological prototypes and engineered structures, the controllable fabrication of complex multi-scale architectures, the long-term stability and safety of bionic materials under realistic service conditions, and the scalability and standardization of fabrication processes. Overall, the contributions in this Special Issue not only enrich the scientific understanding of multi-scale bionic materials but also provide valuable references for their practical applications in biomedicine, sustainable packaging, energy storage, intelligent soft materials, and extreme-environment devices. We hope that this Special Issue will serve as a useful platform for interdisciplinary communication and stimulate further innovation in the design, fabrication, and application of next-generation bionic materials.
